# Antimicrobial Activity of Mesenchymal Stem Cells: Current Status and New Perspectives of Antimicrobial Peptide-Based Therapies

**DOI:** 10.3389/fimmu.2017.00339

**Published:** 2017-03-30

**Authors:** Francisca Alcayaga-Miranda, Jimena Cuenca, Maroun Khoury

**Affiliations:** ^1^Laboratory of Nano-Regenerative Medicine, Faculty of Medicine, Universidad de Los Andes, Santiago, Chile; ^2^Cells for Cells, Santiago, Chile; ^3^Consorcio Regenero, Chilean Consortium for Regenerative Medicine, Santiago, Chile

**Keywords:** mesenchymal stromal cells, mesenchymal stem cells, antimicrobial effect, antibacterial property, AMPs

## Abstract

While mesenchymal stem cells (MSCs)-based therapy appears to be promising, there are concerns regarding possible side effects related to the unwanted suppression of antimicrobial immunity leading to an increased risk of infection. Conversely, recent data show that MSCs exert strong antimicrobial effects through indirect and direct mechanisms, partially mediated by the secretion of antimicrobial peptides and proteins (AMPs). In fact, MSCs have been reported to increase bacterial clearance in preclinical models of sepsis, acute respiratory distress syndrome, and cystic fibrosis-related infections. This article reviews the current evidence regarding the direct antimicrobial effector function of MSCs, focusing mainly on the role of MSCs-derived AMPs. The strategies that might modulate the expression and secretion of these AMPs, leading to enhanced antimicrobial effect, are highlighted. Furthermore, studies evaluating the presence of AMPs in the cargo of extracellular vesicles (EVs) are underlined as perspective opportunities to develop new drug delivery tools. The antimicrobial potential of MSCs-derived EVs can also be heightened through cell conditioning and/or drug loading. Finally, improving the pharmacokinetics and delivery, in addition to deciphering the multi-target drug status of AMPs, should synergistically lead to key advances against infections caused by drug-resistant strains.

## Introduction

Human mesenchymal stem or stromal cells (MSCs) are self-renewing multipotent cells with great potential for regenerative medicine and tissue engineering. MSCs are located within the stroma of the bone marrow (BMSCs) and other organs, including adipose tissue (AT-MSCs), dental pulp (DP-MSCs), postnatal tissues, such as umbilical cord (UC-MSCs) and placenta (PL-MSCs), or menstrual fluid (MenSCs), with each MSCs-population displaying individual differentiation potential and phenotype (1–7). According to the Mesenchymal and Tissue Stem Cell Committee of the International Society for Cellular Therapy (ISCT, www.celltherapysociety.org/), the minimal criteria for defining human MSCs are (a) adherence to plastic surface; (b) specific surface antigen expression (Positive expression of CD105, CD73, and CD90, and lack expression of CD45, CD34, CD14 or CD11b, CD79a or CD19, and HLA-DR); and (c) multipotent differentiation potential to osteoblast, adipocytes, and chondroblasts using standard *in vitro* tissue culture-differentiating conditions (8). Their versatility, homing preference for injured tissue, their immune privileged status, and the lower risk of tumorigenesis render them an interesting instrument in cell-based therapy (7). Due to their differentiation plasticity, immunomodulatory properties, angiogenic modulation, and paracrine support (9–13), MSCs have been investigated in a wide spectrum of disease indications, which is evidenced in the ~500 trials enrolled in the ClinicalTrials.gov database of the NIH (http://www.clinicaltrials.gov/, queried in December 2016).

Despite the demonstrated biologic effect and regenerative properties of MSCs *in vitro* and *in vivo*, the exact knowledge about their mechanism of action is still unknown. However, it is now widely accepted that MSCs exert their effect by paracrine stimulations through the release of small molecules as growth factors, cytokines, and chemokines (14, 15). Although initial clinical results of MSCs-based therapy showed promising outcomes, there are significant concerns that application of MSCs may inadvertently suppress antimicrobial immunity with an increased risk of infection (16). On the other hand, current data suggest that MSCs exert strong antimicrobial effects through indirect and direct mechanisms (16–26). Indirectly, across their role in the host immune response against pathogens, especially in the dynamic coordination of the pro- and anti-inflammatory elements of the immune system (22, 27–29) or increasing the activity of phagocytes (18, 21, 25, 30, 31); and directly, by the secretion of antimicrobial peptides and proteins (AMPs) (19, 20, 23, 24, 26), and also by the expression of molecules such as indoleamine 2,3-dioxygenase (IDO) (16) and interleukin (IL)-17 (32). In fact, MSCs have been reportedly responsible of the bacterial clearance in preclinical models of sepsis (17–20, 22, 25), acute respiratory distress syndrome (ARDS) (21, 26), and cystic fibrosis infection (23).

AMPs or host-defense peptides and proteins are an abundant and diverse group of endogenous molecules that are produced as a first line of defense by all multicellular organisms which have a broad spectrum of antimicrobial and immunomodulatory activities (33). These molecules have selective activity against a wide range of organisms including bacteria, yeasts, fungi, viruses, and even cancer cells (34). So far, MSCs have been found to constitutively express four AMPs: cathelicidin LL-37 (19, 23), human β-defensin-2 (hBD-2) (24), hepcidin (20), and lipocalin-2 (Lcn2) (26), which can be further modulated during infection and inflammation. In fact, it has been demonstrated that, in MSCs, a bacterial preconditioning induces an upregulation of LL-37, hBD-2, and hepcidin (19, 20, 24), while their preconditioning with inflammatory stimuli evoke increasing levels of LL-37 and Lcn2 (23, 26). In different preclinical models, MSCs-derived AMPs have demonstrated to be part of the bacterial clearance effect observed with MSCs treatment, revealing that MSCs can directly enhance the innate immune response to bacterial infection (19, 20, 23, 24, 26).

In this review, we summarize the current evidence regarding the direct antimicrobial effector function of MSCs, focusing mainly in the role of the MSCs-derived AMPs. The different strategies that could modulate the expression and secretion of these AMPs and enhance their antimicrobial effect are emphasized. We also underline the presence of AMPs in the cargo of extracellular vesicles (EVs) present in body fluids and secreted by different type of cells including activated MSCs.

## Antimicrobial Peptides

Antimicrobial peptides are evolutionary conserved gene-encoded small effector molecules (10-150aa) found in organisms from procaryotes to humans (34, 35). Some of these antimicrobial peptides are present constitutively, while others may be induced in response to infection or inflammatory conditions. AMPs display different mechanisms of action (MoA) leading to the elimination of microorganisms, some of which are also dependent on external factors, such as pH, peptide abundance, and salt concentration (33, 36).

Human AMPs interact with different molecular targets either on the cell surface or within the cells. AMPs-mediated cell killing occurs by disrupting membrane integrity, by inhibiting protein, DNA or RNA synthesis, and by interacting with certain intracellular targets (33). In general, AMPs are only effective against one class of pathogen (e.g., bacteria or fungi); however, some AMPs display MoA against several types of microorganisms (37). Importantly, in some specific cases, AMPs can be active against pathogens that are resistant to conventional antibiotics (ABs) as multidrug-resistant bacteria. These advantageous features make AMPs good candidates for drug development, although their clinical and commercial development still needs to overcome challenges, such as route of administration, potential toxicity, stability, and high cost of peptide production (38).

AMPs are also called “host defense peptides,” a terminology that emphasizes their immunomodulatory functions (39). These functions are diverse, specific to the type of AMPs, and include different cytokines and growth factor-like effects that are relevant to both innate and adaptive immune responses (34). Hence, AMPs can also help to resolve inflammation or infections through indirect effects, which act in synergy with the direct antimicrobial activity.

To date, more than 2,700 AMPs of different sources have been registered in the Antimicrobial Peptide Database (APD, http://aps.unmc.edu/AP/main.php; last updated on sixth January 2017) including 115 that are human host defense peptides. In Table 1, we have summarized some representative human AMPs and their characteristics in terms of source, activity, 3D structure, and length.

**Table 1:** **Human selected antimicrobial peptides**.

Name	Source	Activity	3D structure^a^ (35)			Length (aa)	Reference
			α	β	αβ		
Cathelicidin LL-37	Neutrophils, skin monocytes, lymphocytes, MCS, sweat, airway surface liquid, saliva	G, V, F, P, C	√			37	(40)
Dermcidin	Skin, sweat	G, F	√			47	(41)
Granulysin	Cytolitic T and NK cells	G, F, P, C	√			74	(42)
Histatin 5	Saliva	V, F	√			24	(43)
Lactoferricin	Human milk, tears, saliva, bronchial mucus, and seminal plasma	G, V, F, P	√			49	(44)
Lysozyme	Saliva, tears, intestine	G, F	√			130	(45)
Psoriasin/S100A7	Skin, salivary gland, breast	G−	√			101	(46)
α-Defensin HNP-1	Neutrophils, bone marrow	G, V, F, P, C		√		30	(47)
α-Defensin HNP-2	Neutrophils, bone marrow	G, V, F, C		√		29	(47)
α-Defensin HNP-3	Neutrophils, bone marrow	G, V, F, C		√		30	(47)
α-Defensin HNP-4	Neutrophils	G, V, F		√		33	(48)
α-Defensin HD-5	Paneth cells, intestine, female reproductive system	G, V, F		√		32	(49)
α-Defensin HD-6	Paneth cells, intestine	G, V, F		√		32	(50)
Hepcidin 20	Plasma, urine, liver	G, F		√		20	(51)
Hepcidin 25 (LEAP-1)	Plasma, urine/liver heart, kidney, adipose tissue, pancreas and hematopoietic cells, MSCs, myeloid cells (monocytes, macrophages, neutrophils)	G, F		√		25	(51)
Secretory leukoprotease inhibitor (SLPI)	Tears, saliva, airway, gastrointestines, genital tracts	G, V, F		√		102	(52)
RNase 5 angiogenin	Liver, skin, intestine	G+, F			√	125	(53)
Chemokine CCL1	T cells	G			√	73	(54)
Chemokine CCL8	Fibroblasts, endothelial cells	G−			√	75	(54)
Chemokine CCL13	Epithelial cells, mononuclear cells	G-			√	75	(54)
Chemokine CCL20	Skin, B cells, myeloid dendritic cell, memory T cell	G, F, P			√	69	(54, 55)
Chemokine CCL27	Memory T cell	F			√	56	(56)
Chemokine CXCL1	Macrophages, neutrophils, epithelial cells	G			√	73	(54)
Chemokine CXCL10	Monocytes, endothelial cells, fibroblasts	G, F, P			√	77	(57)
β-Defensin hBD-1	Kidney, skin, salivary glands	G, F, C			√	36	(58)
β-Defensin hBD-2	Skin, lung, epithelia, uterus, salivary glands	G, V, F			√	41	(58)
β-Defensin hBD-3	Skin, salivary glands	G, V, F			√	45	(58)
Neutrophil gelatinase-associated lipocalin (NGAL, Lcn2)	Bone marrow, uterus, prostate, salivary gland, stomach, appendix, colon, trachea, lung, small intestine, pancreas, kidney and prostate. MSCs, neutrophils, macrophages, and dendritic cells	G			√	178	(59–63)
RegIIIα	Intestine	G+			√	149	(64)
RNase 7	Urinary tract, respiratory tract, skin	G, F			√	128	(65)

*^a^Data from the APD (http://aps.unmc.edu/AP/database/query_input.php). Antimicrobial activities are annotated as G, bacteria; G+, Gram-positive bacteria only; G−, Gram-negative bacteria only; F, fungi; P, parasites; V, viruses; C, cancer cells*.

Besides their antimicrobial activities, AMPs also show indirect biological effects that may help to eradicate infection (66). In this context, it has been described that AMPs secreted by epithelial cells present a battery of biological features including chemokine and anti-endotoxin activities as well as protease inhibition, bacterial opsonization, and angiogenic properties (66). Specifically, cathelicidins are chemotactic for monocytes, neutrophils, and lymphocytes (67) and it has been demonstrated that LL-37 binds and neutralizes lipopolisacharide (LPS), protecting against endotoxic shock in a mouse model of septicemia (68). Likewise, β-defensins are chemotactic for macrophages, neutrophils, and mast cells, possibly by binding to CCR6 (69). As for hepcidin (70, 71) and Lcn2 (34, 72) they are involved in pathways regulating the availability of iron, a vital element for bacterial growth.

Recently, MSCs have also been described to possess antimicrobial activity, which would be given by antimicrobial peptides or proteins of members of the cathelicidins (19, 23), defensins (24), hepcidin (20), or lipocalin families (26).

Cathelicidins, defensins, and hepcidin are synthesized as pre–pro peptides that are cleaved to release mature AMPs that interact with negatively charged bacterial membrane surface (73). Lipocalins (Lcns) are characterized by their ability to bind small hydrophobic molecules, their binding to specific cell-surface receptors, and their formation of macromolecular complexes (74).

### Cathelicidins

To date, only one cathelicidin gene (CAMP) has been described in mice and human, inducing a protein named CRAMP and LL-37, respectively (75). LL-37 exhibits a broad spectrum of antimicrobial activity, several immunomodulatory effects, anticancer activities, and also chemotactic and pro-angiogenic properties (35, 40). It has been detected in several types of cells, tissues, and fluids, and along with other AMPs, such as β-defensins, plays a central role in mucosal defense (76). An increased or decreased expression of LL-37 has been identified in several diseases, observing elevated levels in inflammatory pathologies like systemic lupus erythematosus and pulmonary diseases (77–79). In contrast, low production of LL-37 is associated with asthma and skin disorders (80–82).

The expression of LL-37 is driven by several stimuli such as inflammatory mediators and microbial structures, and varies depending on cell type (83) (Table 2). Vitamin D3 appears to be the major regulator of LL-37 expression in humans as the CAMP gene encoding LL-37 contains three VDREs (Vitamin D response element) on its promoter. Upregulating the expression of natural LL-37 or developing synthetic mimics might represent a potent new therapeutic option that needs to be carefully evaluated.

**Table 2:** **Factors that can modulate LL-37, hBD-2, hepcidin, and Lcn2 expression**.

LL-37		hBD-2		Hepcidin		Lcn2	
Factor upregulation	Reference	Factor upregulation	Reference	Factor upregulation	Reference	Factor upregulation	Reference
IL-17A (in synergy with vitamin D3)	(84)	LPS	(85)	IL-6, IL-1α, IL-22, oncostatin M	(86, 87)	Anemia	(88)
TNFα, IFNγ	(89, 90)	IL-1, TNFα	(91)	Activin B	(92)	LPS	(62)
Injury, wounding, UVB irradiation	(89, 93)	TLR-2	(94, 95)	Liver metabolic activities	(96)	IL-1α and β, IL-4, IL-17, IL-22, IL-9, TNFα	(97–104)
Sodium butyrate, phenyl butyrate	(105–107)	IL-17	(76)	Oxygenases	(96)	IGF-1, TGFα	(108)
TLR agonists, lithocholic acid, vitamin D receptor agonists	(89, 109–112)	AP-1, MEF	(94, 113)	Small molecule activators of Stat/Smad pathways (genistein)	(114)	Serum, growth factors, phorbol esters, Glucocorticoids (dexamethasone)	(115, 116)
ER stress	(93)	Neutrophil elastase	(117)	BMP6	(118)	3-cis retinoic acid (isotretinoin); 4-HPR	(119, 120)
Short-chain fatty acids, Zn^2+^, lactose	(121–123)	Calcium	(124)	TMPRSS6 siRNA or ASOs	(125)	MK886, nordihydroguaiaretic acid	(126, 127)
BCG	(128)	UV light	(89)	HFE, TfR2	(96)	COX-2 inhibitors, celecoxib-derived PDK1 inhibitors	(126)

**Factor downregulation**	**Reference**	**Factor downregulation**	**Reference**	**Factor downregulation**	**Reference**	**Factor downregulation**	**Reference**

Bacterial exotoxins, *Shigella, Neisseria* infection	(107, 129–131)	Glucocorticoids (dexamethasone)	(76, 124)	Oxidative stress (ROS); hypoxia; heparin; anti-inflammatories (anti-IL-6/IL-6R (siltuximab)/(tocilizumab), anti-TNFα, AG490)	(96, 132–136)	l-Glutamine, *N*-acetylcysteine	(137, 138)
IL-6, IFNγ, calcipotriol	(89, 139)	Calcium quelator	(124)	Matriptase2, s-HJV-Fc, GDF15, erythropoiesis-stimulating agents	(96, 132, 140)	Paricalcitol	(141)
Psychological stress; transmigration across activated endothelium	(142, 143)	Inhibitors of NF-κB and AP-1	(124)	Small molecule inhibitors of the BMPR type I kinase (LDN-193189); anti-BMP6 antibody; HJV ASOs, hepcidin ASOs or siRNA, TfR2 siRNA; PpYLKTK (disruptor of STAT3 dimerization)	(118, 140, 183–187)	EGF, miR-138	(144, 145)

*IL-17A, interleukin-17; TNF, tumor necrosis alpha; IFN, interferon; UV, ultraviolet light; IL-6, interleukin 6; LPS, lipopolysaccharide; IL-1, interleukin 1; IL-22, interleukin 22; TLR, toll-like receptor; BCG, *Mycobacterium bovis* bacillus Calmette–Guérin; AP-1, activator protein 1; MEF, myeloid elf-1-like factor; NF-κB, nuclear factor-κB; BMP6, bone morphogenetic protein 6; TMPRSS6, transmembrane protease serine 6; siRNA, small interfering RNA; HFE, human hemochromatosis protein; TfR2, transferrin receptor 2; s-HJV-Fc, soluble form of hemojuvelin; GDF15, growth differentiation factor 15; BMPR, bone morphogenetic protein receptor; STAT3, signal transducer and activator of transcription 3; ASOs, antisense oligonucleotides; HJV, hemojuvelin; EGF, epidermal growth factor; COX-2, cyclooxygenase-2; PDK1, phosphoinositide-dependent kinase 1; 4-HPR; *N*-(4-hydroxyphenyl)retinamide; ER, endoplasmic reticulum; miR-138, microRNA-138; IGF-1, insulin like growth factor-1; TGFα, transforming growth factor alpha*.

### Defensins

The defensin family of AMPs can be classified as α-defensins, β-defensins, or θ-defensins depending on their cellular origin, gene structure, and connectivity of the cysteine residues in their sequence (75). Defensins play important roles in innate and adaptive immunity against microbial and viral infections and also participate in wound repair, cytokines and chemokine expression, production of histamine, and enhancement of antibody responses (58).

β-Defensins, hBD-1, hBD-2, and hBD-3 are the mainly functional peptides in humans (35) expressed by many epithelial cells, granulocytes, and MSCs (24). Except for hBD-1, their expression is inducible (75), hBD-2 and hBD-3 are induced by pro-inflammatory stimuli or microorganisms (91, 146). hBD-1 and 2 are microbicidal predominantly against Gram-negative bacteria, while hBD-3 is a broad-spectrum antimicrobial peptide. Given their significant antimicrobial and antiviral activity, modulating endogenous defensin production through the use of specific regulatory stimuli makes defensins promising candidates for therapy (Table 2). Direct administration to sites such as the skin or cervico-vaginal mucosa for treatment of wounds or infection is of interest, particularly in conditions where antimicrobial peptide generation is reduced (76).

### Hepcidin

Hepcidin is a natural human host defense peptide, originally found in urine and plasma (147, 148) which is produced mainly by hepatocytes but also by other cells including MSCs and myeloid leukocytes (71).

Hepcidin is known to act as an iron regulatory hormone, as well as it exerts a broad spectrum of antimicrobial activity against fungal species and clinical relevant bacteria such as *Escherichia coli, S. epidermidis, S. aureus*, and group *B streptococci*; however, the role as an antimicrobial peptide remains to be further unraveled for most type of infections (51, 147). Both peptides forms of hepcidin (hep-20 and hep-25) exhibit antimicrobial properties (147); however, hep-20 is not involved in the regulation of iron use (149).

The expression of hepatocyte hepcidin is regulated by iron status through the BMP-SMAD pathway and by inflammation, where the binding of inflammatory cytokines like IL-6 triggers the JAK–signal transducer and activator of transcription 3 (STAT3) signaling pathway (150, 151). An hepcidin-mediated hypoferremia could functions as a host defense mechanism that evolved to restrict iron availability and slow the pathogen growth (70, 71). The enhancement of bactericidal activity at low pH renders these peptides interesting for the development of drugs specifically designed, for example, for bacterial and/or fungal infections occurring in body areas with acidic pH (51, 152). Thus, modulation of the hepcidin pathway (Table 2) has become an interesting target for new therapeutic strategies (140).

### Lipocalins

Lipocalins are a family of small soluble proteins, which are often secreted (88). Lcns functions as transporters binding small organic molecules that have been associated with many biological processes: the immune response, cell growth, proliferation and metabolism, synthesis of prostaglandins, and iron transportation (153). Lcn2 appears to have an important role in innate antimicrobial defense mechanism (154) and in contrast with other AMPs, it exerts an indirect function against pathogens.

Originally, Lcn2 was identified as a component of neutrophil granules; however, it was later shown to be also expressed in various cells such as macrophages, adipocytes, MSCs, and epithelial cells in response to inflammatory conditions (26, 115, 153, 155) (Table 2). The antimicrobial role is given by its ability to sequesters bacterial iron chelators, called siderophores, that consequently prevent the iron transfer to bacteria and arrested their growth (bacteriostatic effect) (34, 72). This function was demonstrated using mice genetically lacking Lcn2 that showed a marked increase in mortality in an *E. coli*-induced sepsis model (155, 156). Other studies revealed that Lcn2 also binds other types of siderophores, such as bacillibactin (*B. anthracis*) (157) and carboxy-mycobactin (mycobacteria) (158). Lcn2 is one of the most commonly studied novel biomarkers (159) and has emerged as a promising biomarker for different pulmonary diseases (97), kidney injury (160–163), liver failure (164), and tumorigenesis (144). Thus, the clinical relevance of Lcns is rapidly increasing even though the exact mechanisms behind their functions remain to be elucidated.

## Antimicrobial Effector Function of MSCs

### *In Vitro* Studies

Most of the data about the antimicrobial properties of MSCs have been obtained from *in vitro* studies with bacteria, although little data exist about the effect of MSCs on viral, fungal, and parasite pathogens. For both unstimulated and stimulated MSCs, a direct antimicrobial effect has been described (Table 3).

**Table 3:** **Summary of direct antimicrobial effects of MSCs on bacterial, fungal, parasite, and viral pathogens**.

MSCs stimuli	Activity	Mechanism of action	Reference
Bacteria-stimulated BMSCs	Growth inhibition of Gram-negative (*Escherichia coli, Pseudomonas aeruginosa*) and Gram-positive (*S. aureus*)	↑ LL-37	(19)
Unstimulated and bacteria-stimulated MenSCs and BMSCs	Growth inhibition of a mix of bacteria	↑ Hepcidin	(20)
Unstimulated and stimulated BMSCs and AT-MSCs with inflammatory stimuli	Growth inhibition of Gram-negative (*P. aeruginosa*) and Gram-positive (*S. aureus*; *S. pneumoniae*)	↑ LL-37	(23)
Bacteria-stimulated UC-MSCs	Growth inhibition of Gram-negative (*E. coli*)	↑ hBD-2	(24)
Stimulated muBMSCs with inflammatory stimuli	Inhibition of bacteria growth	↑ Lipocalin-2, ↑ Phagocytic activity	(26)
IFNγ-stimulated BMSCs	Growth inhibition of Gram positive (*S. aureus*; *S. epidermidis*; *E. faecium*; *Group B streptococci*) and parasite (*Toxoplasma gondii*), and reduction in virus replication (CMV and HSV-1)	↑ IDO	(16)
muBMSCs producing IL-17	Growth inhibition of *Candida albicans*	↑ IL-17	(32)

*MSCs, human mesenchymal stem cells; BMSCs, bone marrow MSCs; muBMSCs, murine BMSCs; MenSCs, menstrual MSCs; AT-MSCs, adipose tissue MSCs; UC-MSCs, umbilical cord MSCs; IFNγ, interferon gamma; IL, interleukin; hBD-2, human β-defensin-2; IDO, indoleamine 2,3-dioxygenase; CMV, cytomegalovirus; HSV-1, herpes simplex virus type-1*.

The antimicrobial efficacy of MSCs mediated by AMPs has been described for different sources of stromal cells, although different MoA and antibacterial range have been reported for them. Probably, these variations in the antimicrobial spectrum of MSCs might be a specific response of MSCs to produce the most effective AMPs against a specific type of pathogen challenge. A summary of the types of AMPs detected and not detected in the different sources of MSCs are shown in Table 4. Likewise, the data available to date suggest notable species-specific difference between murine and human MSCs with regard to the MoA of the antimicrobial effector function of MSCs (16).

**Table 4:** **Summary of AMPs types described in MSCs according to their source of origin**.

Type of MSCs	Types of AMPs							
	LL-37	Hepcidin	β-Defensins			SP-D	Lipocalin-2	Reference
			hBD-1	hBD-2	hBD-3			
BMSCs	√	√	x	√/x	x	x	x	(19, 20, 23)
muBMSCs	–	–	–	–	–	–	√	(26)
AT-MSCs	–	–	–	–	–	–	–	(23)
MenSCs	x	√	x	x	x	–	–	(20)
UC-MSCs	x	–	–	√	–	–	x	(24)

*MSCs, human mesenchymal stem cells; BMSCs, bone marrow MSCs; muBMSCs, murine BMSCs; MenSCs, menstrual MSCs; AT-MSCs, adipose tissue MSCs; UC-MSCs, umbilical blood cord MSCs; hBD, human β-defensin; SP-D, surfactant protein-D; √, detected; x, not detected; –, not determined*.

BMSCs are the most studied source regarding the intrinsic antimicrobial ability of MSCs. In humans, the antimicrobial effect of BMSCs is mediated by LL-37 (19, 23) and hepcidin (20).

These AMPs have been detected in both unstimulated and stimulated-BMSC cultures. Respect to LL-37, BMSCs as well as their conditioned medium has demonstrated the property to inhibit the bacterial growth of *E. coli, Pseudomonas aeruginosa, S. aureus*, and *S. pneumonia* (19, 23). In the study performed by Krasnodembskaya et al. (19), the authors showed that BMSCs are able to inhibit bacterial growth directly, also by means of conditioned culture medium, but only when BMSCs were previously challenged with bacteria. They also showed that BMSCs produce and secrete inducible quantities of LL-37, responsible for the inhibition of bacterial growth of *E. coli* and *P. aeruginosa in vitro*. In contrast, hBD-2-3, Lcn2, and surfactant protein D (SP-D) were negative in BMSCs or expressed very low protein levels, which were insufficient to elicit an antibacterial effect. Likewise, these cells exhibit direct antimicrobial activity against *S. aureus*, through a contact-independent mechanism and mediated in part by the release of LL-37. Recently, Sutton et al. (23). demonstrated that BMSCs constitutively secrete LL-37, which exert a potent antimicrobial effect *in vitro* against *P. aeruginosa, S. aureus*, and *S. pneumonia*, controlling the rate of bacterial growth and transition into colony forming units. These data suggest that BMSCs supernatants can be useful as an adjunct treatment to conventional ABs. Interestingly, the levels of LL-37 were enhanced after the preconditioning with inflammatory stimulus, as IFNγ, IL-1β, or IL-12. However, in line with previous report (19, 20), the levels of hBD-2-3 were not detected in unstimulated nor stimulated BMSCs (23). Furthermore, the levels of LL-37 were detected in BMSCs only when the cell growth was performed under FBS (fetal bovine serum) condition, since the serum-free or platelet lysate conditions reduce the antimicrobial efficacy of BMSCs and their production of LL-37 (23). Conversely with the aforementioned data, in which expression of the antimicrobial peptide LL-37 was detected constitutively in BMSCs or following stimulation with *E. coli* or inflammatory stimulus, data from our research group did not show any basal or induced expression of LL-37 under both bacterial mixture and LPS stimulations in BMSCs. However, we detected in bacteria-stimulated BMSCs the expression of hepcidin, which was involved in the antimicrobial effect of MSCs both direct and in conditioned medium (20).

The antimicrobial properties of MSCs is not only limited to AMPs activity. In fact, upon stimulation with inflammatory cytokines, BMSCs through a substantial increase in IDO expression, exhibit a cell autonomous, broad-spectrum antimicrobial effector function directed against clinically relevant bacteria (*S. aureus; S. epidermidis; E. faecium; Group B streptococci*), protozoal parasites (*Toxoplasma gondii*), and viruses (CMV and HSV-1) (16). Also, it has been reported that IL-17-positive murine(mu)BMSCs exert a potent antifungal activity with respect to IL-17-negative or bulk muBMSCs. The growth inhibition of *Candida albicans* is mediated by IL-17 in a dose-dependent manner, while anti-IL-17 antibodies partially reduce anti-*C. albicans* effect of muBMSCs (32).

Potent antimicrobial effects of muBMSCs have also been described on *E. coli* and *C. albicans* but with a different MoA with respect to their human counterpart. As reviewed by Balan et al. (165), no data indicate secretion of LL-37 by muBMSCs, but the preconditioning with *E. coli* significantly increases the production of the antimicrobial protein Lcn2 (26). Furthermore, in contrast to BMSCs of human origin, muBMSCs fail to express IDO even after stimulation with inflammatory cytokines such as IFNγ, tumor necrosis alpha, and IL-1β, and they consequently do not inhibit bacterial growth (16). However, it has been reported that unstimulated muBMSCs directly exhibit phagocytic activity for *E. coli* or *S. aureus* in a cell dose-dependent manner (25), although further studies are required to confirm these claims.

More recently, menstrual fluid-derived MSCs (MenSCs) have emerged as an attractive alternative for cell therapy since they are isolated in a non-invasive manner with the possibility of periodical collections from the same donor, ensuring high amounts of cells at low culture passages and from the same genetic background (2, 6). Our group has recently shown that MenSCs have an important antimicrobial effect mediated by hepcidin, both directly and through the peptide present in their conditioned medium. Polybacterial stimulation significantly increased the expression of hepcidin in MenSCs, whereas other antimicrobial peptides such as LL-37 and hBD-1-2-3 remained below the limit of detection (20). Interestingly, the downregulation of hepcidin through hypoxic condition resulted in the loss of the antimicrobial property of MenSCs, indicating a hepcidin-dependent mechanism (20). Therefore, culture condition need to be carefully considered when expanding MenSCs as any modification in the oxygen levels can affect their biological activities and consequently, their antimicrobial potential. In that perspective, the effect of hypoxia on MenSCs functions could be considered as a negative regulator of their antimicrobial activity (20) but is contrasted with a positive effect on their angiogenic property (2). Nevertheless, the extend of the effect of this type of conditioning is largely dependent on the source of MSCs, as an example, it has been observed that the immunosuppressive capacity of AT-MSCs remains unchanged under hypoxic conditions (166).

Sung et al. (24) described that the growth of *E. coli in vitro* was significantly inhibited by UC-MSCs or their conditioned medium after bacterial exposure. In microarray analysis performed to detect gene expression changes in MSCs responsible for their antibacterial action, a significant upregulation of toll-like receptor (TLR)-2 and TLR-4, and hBD-2 were identified in UC-MSCs after *E. coli* exposure, suggesting that the antimicrobial effects of MSCs might be mediated by the secretion of hBD-2 via TLR signaling pathway. However, the increased hBD-2 level and the *in vitro* antibacterial effects of MSCs were abolished by specific antagonist or by silencing of the TLR-4, but not TLR-2. The effect was restored by hBD-2 supplementation, indicating that hBD-2 secreted by MSCs only via TLR-4 mediates the antibacterial effects. In this study, the gene expression profiles of other antimicrobial proteins, such as LL-37 (19, 23) or Lcn2 (26), were not significantly upregulated.

AT-MSCs or their conditioned medium have also shown an antimicrobial effect. Sutton et al. (23) report that the conditioned medium of AT-MSCs decreased the *P. aeruginosa* growth rate to levels comparable with the AB geneticin, a broad-spectrum aminoglycoside AB. Importantly, the combination of AT-MSCs with geneticin showed a potent antimicrobial effect against *P. aeruginosa*, revealing that this type of stromal cells have an AB-enhancing effect.

### *In Vivo* Studies

The *in vitro* data demonstrating the antimicrobial effect of MSCs have also been supported by *in vivo* studies. MSCs from different sources or origins have the capability to reduce the burden of pathogens in different preclinical models, independently of the route of administration, doses, or time of injections, having been shown in blood, spleen, peritoneum, lung, and bronchoalveolar lavage (BAL) fluid (17–23, 25, 26, 30). In Table 5, we summarize the current *in vivo* data regarding antimicrobial effector function of MSCs mediated by AMPs.

**Table 5:** **Summary of studies reporting direct antibacterial effect of MSCs by AMPs**.

Model	MSCs type/route	Pre-conditioning	*In vitro* findings	*In vivo* findings	Reference
Mouse model of *Escherichia coli* pneumonia (C57BL/6 mice)	BMSCs/it	*E. coli*	↑ LL-37	↓ Bacterial growth in lungs and in BAL fluid	(19)
			↑ Inhibition of bacterial growth (*E. coli, Pseudomonas aeruginosa, S. aureus*)		
Mouse model of CLP-induced sepsis (C57BL/6j mice)	MenSCs/ip	Unstimulated and stimulated with bacterial mix	↑ Hepcidin ↑ Inhibition of bacterial growth	↑ Survival	(20)
				↓ Lung injury	
				↑ Bacterial clearance in the peritoneal fluids and blood	
				Modulation of inflammatory response	
Mouse model of cystic fibrosis (*CFTR^tmlKth^* mice)	BMSCs AT-MSCs/retro-orbital sinus	Unstimulated and stimulated with IFNγ, IL-1β, or IL-12	↑ LL-37	↓ Bacterial growth in BAL fluid	(23)
			↑ Inhibition of bacterial growth (*P. aeruginosa*; *S. aureus*; *S. pneumoniae*)		
Mouse model of *E. coli* pneumonia (ICR mice)	UC-MSCs/it	*E. coli*	↑ hBD-2 ↑ Inhibition of bacterial growth (*E. coli*)	↓ Alveolar congestion, hemorrhage, neutrophil infiltration, and wall thickening	(24)
				↓ Bacterial growth, protein concentrations and cytokine levels (IL-1α, IL-1β, IL-6, and TNFα) in BAL fluid	
				↑ hBD-2 in BAL fluid	
Mouse model of Gram-negative pneumonia (C57BL/6 mice)	muBMSCs/it	LPS and TNFα	↑ Lipocalin-2 ↑ Phagocytic activity	↑ Survival	(26)
				↓ Lung injury	
				↑ Bacterial clearance from the alveolar space	
				↓ MIP-2, TNFα, and MPO in BAL fluid	
				↑ Lipocalin-2 in BAL fluid	

*MSCs, mesenchymal stem cells; BMSCs, bone marrow MSCs; muBMSCs, murine BMSCs; MenSCs, menstrual MSCs; AT-MSCs, adipose tissue MSCs; UC-MSCs, umbilical cord MSCs; it, intratracheal; ip, intraperitoneal; CLP, cecal ligation and puncture; BAL, bronchoalveolar lavage; CM, conditioned media; IFNγ, interferon gamma; IL, interleukin; hBD-2, human β-defensin-2; TNFα, tumor necrosis factor alpha; LPS, lipopolysaccharide; MIP-2, macrophage inflammatory protein 2; MPO, myeloperoxidase*.

In an immunocompetent model of pneumonia by *E. coli*, the treatment with 1 × 10^6^ BMSCs after 4 h of *E. coli* instillation induced a sharp reduction in total bacterial counts in lung homogenates (LH) and BAL fluid compared with vehicle (19). Total BAL cell counts and absolute neutrophil counts were also lower in the BMSCs-treated group, suggesting that bacterial clearance in the BAL of BMSC-treated mice did not primarily depend on the recruitment of immune cells. In fact, the administration of BMSCs together with neutralizing anti-LL-37 antibody resulted in a 10-fold increase in bacterial number both in LH and BAL, revealing that LL-37 effect is needed for the antimicrobial activity of BMSCs *in vivo* (19). These results are in line with Sung’s work (24), where in the same model, the treatment with 1 × 10^5^ UC-MSCs administered intratracheally after 3 h of *E. coli* instillation provoke a downregulation of the inflammatory response and enhanced bacterial clearance, increased hBD-2 secretion in BAL fluid and the resultant protection against *E. coli*-induced pneumonia. Since hBD-2 secreted by MSCs are via the TLR-4 pathway, the antibacterial effects of UC-MSCs were abolished with TLR-4 siRNA transfection of UC-MSCs. Likewise, the detection of hBD-2 in BAL fluid, suggests that hBD-2 secreted by transplanted UC-MSCs plays a pivotal role in mediating the *in vivo* antibacterial effects of UC-MSCs (24). Similarly, another study with similar preclinical model setting described that the concentration of the antimicrobial molecule Lcn2 in BAL fluid is increased after the transplantation of muBMSCs; and the blocking of Lcn2 by a monoclonal antibody results in the elimination of the antibacterial effect of MSCs treatment *in vivo* (26).

In immunocompetent mice with a polymicrobial sepsis induced by cecal ligation and puncture (CLP), the administration of MSCs, either of human or murine origin, induced a reduction of the burden of pathogens and also a significant improvement in the survival rate (17, 20, 22, 25, 30). Our group has recently shown that the administration of 7.5 × 10^5^ MenSCs 3 h after CLP-induced sepsis is able to reduce the animal mortality regulating different aspects of the septic process, such as the organ dysfunction, modulation of the inflammatory response without severe immunosuppression, and promotion of bacterial clearance in blood, in a similar degree to the present standard of treatment with AB therapy (20). Notably, the synergism between MenSCs + AB resulted in the highest improvement of survival (20). Although the *in vivo* role of hepcidin was not evaluated, the *in vitro* data suggest a potential antibacterial effect of hepcidin in the resolution of the bacteremia. In line with these data, Gonzalez-Rey et al. (17) described that AT-MSCs-treated septic mice have lower peritoneal bacterial counts than untreated mice, suggesting that AT-MSCs could promote bactericidal activities by themselves or on other cell types. Concordantly, Mei et al. (25) reported that 2.5 × 10^5^ muBMSCs administered 6 h after CLP-induced sepsis results in a higher bacterial clearance in part due to enhanced phagocytotic activity of the host immune cells.

Cystic fibrosis is a genetic disease in which the battle between pulmonary infection and inflammation becomes the major cause of morbidity and mortality. In a mouse model of cystic fibrosis infected with *P. aeruginosa* and *S. aureus*, the administration of 1 × 10^6^ BMSCs or its conditioned medium results in a reduction of the CFU of these two pathogens in BAL fluid. Although the role of the peptide LL-37 was not evaluated *in vivo*, the *in vitro* data showing the constitutive or stimulated expression of LL-37 suggest a potential role for this peptide in the reduction of bacterial growth *in vivo* (23).

## Are Antimicrobial Extracellular Vesicles, the Next Big Thing for Infectious Diseases?

Extracellular vesicles released by cells act as key agents in intercellular communications through transfer of information via their cargo, which includes proteins, DNAs, and RNAs (167). The active biological role of cell-derived vesicles in pathological conditions such as ischemic heart disease, kidney injury, and wound healing have been shown to share many similar therapeutical features of their parent cells (15). While EVs [mainly microvesicles (MVs) and exosomes] are gaining momentum in the regenerative medicine field as a putative surrogate to cell-based therapeutics (168) their application in infectious diseases still requires further exploration.

Recently, AMPs were identified in different EVs isolated from body fluids and selective enrichment was related to specific diseases. Hiemstra et al. (169) reported that exosomes isolated from normal human urine contain innate immune proteins that include antimicrobial agents. The analyzed list included lysozyme C, dermcidin, mucin-1, calprotectin, and myeloperoxidase, all known for their bactericidal effect. The antimicrobial activity of urinary exosomes was determined through their *in vitro* incubation with *E. coli*, reporting that these exosomes were able to induce bacterial lysis and inhibit their growth *in vitro*, observations that reinforce the claim that exosomes are innate immune effectors that contribute to host defense. Similarly, exosomes isolated from nasal lavage fluids were shown to contain proteins with immune-related functions relevant in the first line of defense against pathogens and allergens. Thus, the analysis of the alterations in the exosomes proteome as a result of a chronic airway inflammation revealed that serum-associated proteins and mucins were more abundant in exosomes from subjects with airway diseases compared to healthy subjects while proteins with antimicrobial functions and barrier-related proteins had decreased expression (170). Also, biliary and intestinal epithelium luminal release exosomes that are increased following infection by a protozoan parasite, carrying AMPs of epithelial cell origin, including LL-37 and hBD-2. Activation of TLR-4 signaling enhances exosome-mediated shuttle of epithelial AMPs from the gastrointestinal epithelium, revealing a new arm of mucosal immunity relevant to antimicrobial defense (171).

As discussed in this review, MSCs exert strong antimicrobial effects through paracrine release of several antimicrobial factors. However, future experimental designs are still required to demonstrate whether some of these effects are mediated by EVs.

### Potential Antibacterial Activity of EVs Released from MSCs

Recent studies have ignited significant interest on EVs released by MSCs accounting, at least in part, on their paracrine effect resulting in a horizontal transfer of the nucleic acids and proteins between the injured cells and MSCs. This cell-to-cell interaction is doubt to be bi-directional, resulting in reprogramming of their secretome to respond to specific need of the tissue by transferring to the injured cells, factors restraining injury, and leading to tissue regeneration. MSCs-derived EVs have been implicated in the tissue restoring effects of MSCs including, anti-apoptotic (172), wound healing (173), and anti-tumoral activities (168).

**Figure 1:**
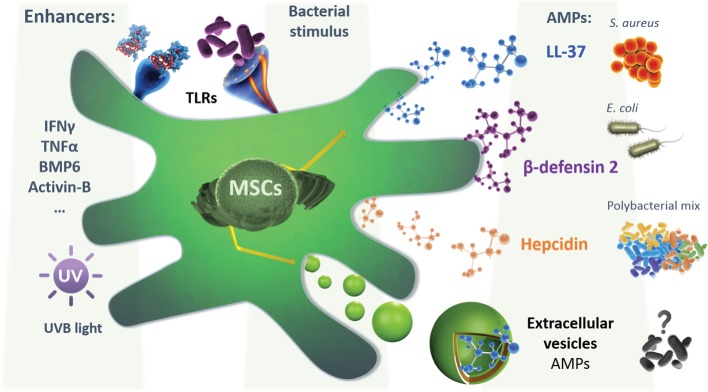
**A schematic representation depicting the mesenchymal stem cells (MSCs) secretion of different AMPs, following different hypothetical preconditioning to enhance their expression, secretion, or encapsulation in extracellular vesicles (EVs), based on the known regulation of the expression of each AMPs**. Also, the bacterial or inflammatory stimulation is shown. LL-37 secretion was shown to possess a bactericide effect on both *S. aureus* and *Escherichia coli*, while the β-defensin-2 effect was demonstrated in *E. coli* alone. MSCs isolated from menstrual fluids were able to secrete hepcidin that was shown to inhibit the growth of a polybacterial mix isolated from mice microflora. Extracellular vesicles can also be secreted by MSCs and possibly contain active agents with potential antimicrobial effect. This will require further investigation in the future. Abbreviations: IFNγ, interferon gamma; TNFα, tumor necrosis factor alpha; BMP6, bone morphogenetic protein 6; AMPs, antimicrobial peptides.

In the last few years, the beneficial role of MSCs-derived EVs has been described in several preclinical models of inflammatory/infectious diseases. Zhu et al. (174) reported that MVs released by BMSCs were therapeutically effective following *E. coli* endotoxin-induced acute lung injury (ALI) in mice, in part through the transfer of keratinocyte growth factor (KGF) mRNA from the MVs to the injured alveolar epithelium and lung endothelium. In line, Monsel et al. showed that the administration of MVs secreted by BMSCs improved survival, and decreased the influx of inflammatory cells and bacteria in a bacterial pneumonia mouse model. The antimicrobial effect of BMSCs-derived MVs was in part through enhancement of monocyte phagocytosis of bacteria, which could be further increased by pre-stimulation of BMSCs with a TLR-3 agonist before the release of MVs (175). However, it remains to be explored whether MVs released from MSCs conserve the cell antimicrobial effect through their AMPs content. Future experimental designs are required to explicitly address their antimicrobial relevance in the previously evaluated infectious disease models. Furthermore, the exposure of MSCs to conditions that are known to upregulate the expression of AMPs such as cytokines (IFNγ, TNFα, IL-22, etc.), growth factors, and UV exposure should be considered (Figure 1). Additionally, the effect of the conditioning on increasing the MVs cargo content with AMPs will also need to be evaluated. Nevertheless, in other applications, it has been demonstrated that MVs can transport drugs to target sites and maintain a higher drug concentration than conventional dosage forms. Tang et al. engineered drug-packaging MVs by incubating cells with chemotherapeutic agents. These hybrid MVs were used to effectively kill tumor cells in murine tumor models without typical side effects (176). More recently, another group assessed the feasibility of an exosome-based drug by loading a chemotherapeutic agent through sonication to treat multidrug-resistant cancer. Interestingly they observed a 50-fold increase in the cytotoxicity in drug-resistant cancer cells (177). This is a relevant outcome as it limits the side effect related to the conventional administration of chemotherapies and advance a novel and sustained drug release method.

Similar drug loading strategy could be evaluated based on AMPs packaging in EVs. The rationale for EVs loaded with AMPs is the possibility to enhance their therapeutic potential through increasing their concentrations at the site of infection and/or by reducing the toxicity of ABs.

## Trends, Future Perspectives, and Challenges

Aside from the direct administration of synthetic or natural AMPs through drug or MSCs infusion, there are several ongoing clinical studies aimed at modulating the expression of AMPs by the endogenous cells in the body, through dietary nutrients and vitamins, to boost the innate immune response (178). Indeed, Vitamin D3 has been shown to directly regulate expression of the human antimicrobial peptides LL-37 and hBD-2 (179, 180). However, a recent pilot study in mechanically ventilated ICU patients showed that high dose of vitamin D3 was associated with decreased hospital length of stay, but no statically significant change in plasma LL-37 concentrations or other clinical outcomes (181). While the stimulatory effect of Vitamin D has been described for the osteogenic potential of MSCs (182), future experiments are required to address their effect on the expression of AMPs both *in vitro* and *in vivo*. This could represent an alternative approach by stimulating the endogenous MSCs to secrete higher amount of AMPs.

The urgent need for new strategies has become evident due to increasing drug resistance. Discovering new classes of antimicrobials or AMPs is a very promising approach but it might not be the only strategy required for defeating this threat. In fact, enhancing AMPs delivery, bioavailability, liberation, absorption, half-life while reducing their clearance and side effect can also denote an additional booster for overcoming infections.

Mesenchymal stem cells present desirable features to improve the pharmacokinetics of natural AMPs, their stem cell-related “arsenal” includes

(a) *Sensors*, able to detect and react following an infection signal, triggering an immediate mobilization and a migration shift toward the site of infection.
(b) *Bioactive pumps*, able to control on-demand the continuous release of AMPs in the site of infection.
(c) *Watchtowers*, able to release specific combinations of AMPs depending on the stimulus.

Standpoint efforts focusing on enhancing these MSC features through genetic modifications or preconditioning, in addition to deciphering the multi-target drug status of AMPs (indirect and direct targets), should synergistically lead to key advances against infections caused by drug-resistant strains. Nonetheless, despite these advantageous features, there are still some challenges hindering their applications, such as high production costs (when compared to generic ABs), in addition to storage problems including “off-the-shelf” and ready-to-use dosage shortage.

New perspective tools based on unmodified or drug-loaded EVs are looming in the horizon. These thriving alternatives need further development and investigation to achieve maximum killing efficiency, less side effects, and null drug-resistant infections. Indeed, the prospect of using concentrations below the minimum inhibitory concentration might contribute to limit the emergence of AB resistance due to drug-resistant strains. However, one should keep in mind that the mechanism and effect of EVs on extracellular bacterial clearance might be different in comparison to intracellular bacteria, and this will require additional consideration in the experimental design. The actual trend in the field positions MSCs-derived EVs (with or without drug loading), as important candidates to be exploited as novel “ready-to-inject” biologics, in infectious diseases as an alternative to stem cell-based therapy.

## Conclusion

Mesenchymal stem cells exert strong antimicrobial effects through indirect and direct mechanisms, partially through the secretion of AMPs. The bacterial clearance observed in preclinical models including sepsis, ARDS, and cystic fibrosis infection, can be further enhanced following specific preconditioning protocols. We propose possible strategies to increase the expression and secretion of these AMPs to enhance their antimicrobial effect. These conditions include the exposure of MSCs to cytokines, growth factors, and UVB light. Furthermore, studies evaluating the presence of AMPs in the cargo of EVs will represent an opportunity to develop new drug delivery tools. The potential antimicrobial activity of MSCs-derived EVs can also be heightened through cell conditioning and/or drug loading. Finally, the enhanced antimicrobial potential of MSCs through AMPs secretion or encapsulation present an important potential to be exploited as an improved therapeutic approach for infectious diseases.

## Author Contributions

All authors listed have made substantial, direct, and intellectual contribution to the work and approved it for publication.

## Conflict of Interest Statement

MK is the chief science officer of Cells for Cells and Consorcio Regenero. FA-M and JC received stipends from Cells for Cells. The reviewers HG and FC and handling Editor declared their shared affiliation, and the handling Editor states that the process nevertheless met the standards of a fair and objective review.
